# High Prevalence and Diversity of Caliciviruses in a Community Setting Determined by a Metagenomic Approach

**DOI:** 10.1128/spectrum.01853-21

**Published:** 2022-02-23

**Authors:** Xaira Rivera-Gutiérrez, Patricia Morán, Blanca Taboada, Angélica Serrano-Vázquez, Pavel Iša, Liliana Rojas-Velázquez, Horacio Pérez-Juárez, Susana López, Javier Torres, Cecilia Ximénez, Carlos F. Arias

**Affiliations:** a Instituto de Biotecnología, Universidad Nacional Autónoma de México, Cuernavaca, Mexico; b Unidad de Investigación en Medicina Experimental, Facultad de Medicina, Universidad Nacional Autónoma de México, Mexico City, Mexico; c Unidad de Investigación Médica en Enfermedades Infecciosas y Parasitarias, Hospital de Pediatría, Centro Médico Nacional Siglo XXI, Instituto Mexicano del Seguro Social, Mexico City, Mexico; US Food and Drug Administration

**Keywords:** calicivirus, community setting, next-generation sequencing, early life

## Abstract

We recently carried out a metagenomic study to determine the fecal virome of infants during their first year of life in a semirural community in Mexico. A total of 97 stool samples from nine children were collected starting 2 weeks after birth and monthly thereafter until 12 months of age. In this work, we describe the prevalence and incidence of caliciviruses in this birth cohort. We found that 54 (56%) and 24 (25%) of the samples were positive for norovirus and sapovirus sequence reads detected by next-generation sequencing, respectively. Potential infections were arbitrarily considered when at least 20% of the complete virus genome was determined. Considering only these samples, there were 3 cases per child/year for norovirus and 0.33 cases per child/year for sapovirus. All nine children had sequence reads related to norovirus in at least 2 and up to 10 samples, and 8 children excreted sapovirus sequence reads in 1 and up to 5 samples during the study. The virus in 35 samples could be genotyped. The results showed a high diversity of both norovirus (GI.3[P13], GI.5, GII.4, GII.4[P16], GII.7[P7], and GII.17[P17]) and sapovirus (GI.1, GI.7, and GII.4) in the community. Of interest, despite the frequent detection of caliciviruses in the stools, all children remained asymptomatic during the study. Our results clearly show that metagenomic studies in stools may reveal a detailed picture of the prevalence and diversity of gastrointestinal viruses in the human gut during the first year of life.

**IMPORTANCE** Human caliciviruses are important etiological agents of acute gastroenteritis in children under 5 years of age. Several studies have characterized their association with childhood diarrhea and their presence in nondiarrheal stool samples. In this work, we used a next-generation sequencing approach to determine, in a longitudinal study, the fecal virome of infants during their first year of life. Using this method, we found that caliciviruses can be detected significantly more frequently than previously reported, providing a more detailed picture of the prevalence and genetic diversity of these viruses in the human gut during early life.

## INTRODUCTION

Human caliciviruses are considered the major cause of nonbacterial sporadic and epidemic gastroenteritis in children less than 5 years of age, particularly in countries where rotavirus vaccination has been successfully implemented and the number of rotavirus infections have been reduced (1, 2). These viruses belong to the family *Caliciviridae*, have a positive-sense, single-stranded RNA genome, and include two genera, *Norovirus* and *Sapovirus* (3). While noroviruses are increasingly recognized among the leading causes of sporadic infections and outbreaks in the pediatric population (2), sapoviruses are usually related to sporadic gastroenteritis, although they have also been described in diarrheal outbreaks (4, 5). Both virus species are also commonly reported as a cause of symptomatic or asymptomatic carriage in community settings (2, 6). Currently, noroviruses are classified into 10 genogroups (GI to GX), 5 of which have been found to infect humans, GI, GII, GIV, GVIII, and GIX (3). Worldwide, the two most frequently detected genogroups are GI (9 genotypes) and GII (22 genotypes), with GII.4 being the most predominant genotype (7). However, sapoviruses are classified into at least 15 genogroups (GI to GXV), of which GI (7 genotypes), GII (8 genotypes), GIV (1 genotype), and GV (2 genotypes) infect humans (8).

Human caliciviruses have acquired relevance in public health during the last 3 decades as etiological agents of acute gastrointestinal infections in children. A large number of studies have been conducted, most of them in hospitalized children where both norovirus and sapovirus have been frequently detected by reverse-transcription-PCR (RT-PCR), although in most cases the search has been directed to norovirus (9). Asymptomatic norovirus infections have been found to be common in children under 5 years old, with prevalence ranging from 3.5% to 13.3% (9). In Mexico, a study carried out in children under 2 years old in a periurban community found 19% of norovirus prevalence in diarrheal stool samples and 7% in nondiarrheal samples (10). In a similar study carried out in asymptomatic children, a norovirus prevalence of 29.8% was reported (11).

We recently conducted a random, next-generation sequencing metagenomic study in stools of infants in a semirural community of Mexico to determine the composition and dynamics of the fecal virome of children during their first year of life (12, 13). Samples from nine children were collected starting 2 weeks after birth and monthly thereafter until 12 months of age. Considering the relevance of caliciviruses as infectious agents in children and their frequent detection in infant fecal samples, we analyze in this report the calicivirus component of the virome as part of a larger study that will be published elsewhere. We found a high prevalence of both norovirus and sapovirus in the stool samples early in life and throughout the first year of life; of interest, all children remained asymptomatic during the year of study.

## RESULTS

### Study population and sample analysis.

**FIG 1:**
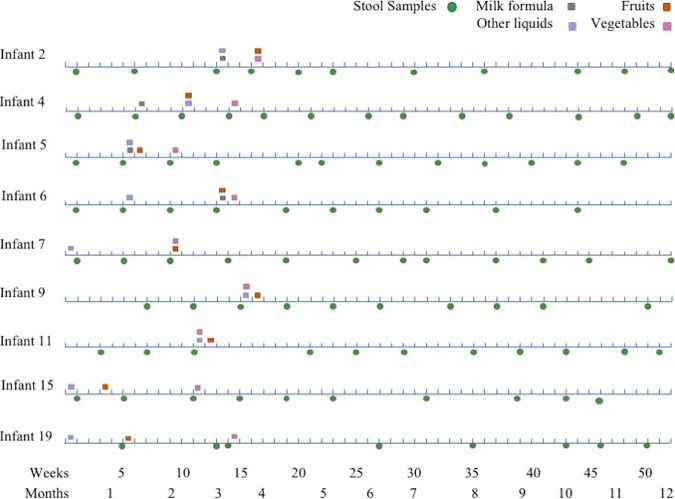
Timeline of stool sample collection. The date of sample collection is shown with a green circle. The week when the children started to ingest liquids or foods different from breastmilk is indicated. Squares represent the start of ingestion of different types of food.

This study was conducted in Xoxocotla, Morelos, a semirural town in the central region of Mexico (12). Stool samples from 9 apparently healthy infants were collected monthly during the first year of life, starting 2 weeks after birth and until 12 months of age (Fig. 1). All infants remained without gastrointestinal symptoms during the period of the study; growth, food ingestion, socioeconomic data, diseases, and other characteristics were recorded in questionaries applied periodically (Table 1).

**TABLE 1:** General information of infants and their living conditions

		General information					Living conditions								
Infant	Birth date (day/mo/yr)	Days after birth (first sample)	Delivery	Sex^*a*^	Mean wt percentile	Age in mo of introduction of other liquids	Overcrowding	Water storage	Water consumption	Sewer system	Cooking facilities	Floor material	Presence of cockroaches	Presence of rodents	Domestic animals
2	04/03/15	15	Vaginal	M	50th	4	Yes	Container	Tap water	No	Stove	Soil	No	No	Yes
4	15/04/15	15	Cesarean	M	15th	3.5	No	Container	Tap water	No	Wood	Concrete	Yes	Yes	Yes
5	25/04/15	16	Cesarean	F	25th	2	No	Cistern	Bottled	Yes	Stove	Concrete	Yes	No	Yes
6	09/06/15	13	Cesarean	M	50th	2	Yes	Container	Tap water	Yes	Wood	Concrete	No	No	Yes
7	23/06/15	16	Vaginal	F	25th	1	Yes	Cistern	Bottled	No	Wood	Soil	No	No	Yes
9	28/11/15	53	Vaginal	F	75th	3.5	Yes	Cistern	Bottled	No	Stove	Soil	Yes	Yes	Yes
11	16/10/15	26	Cesarean	F	50th	5	No	Container	Tap water	No	Wood	Soil	Yes	Yes	Yes
15	29/12/15	8	Vaginal	F	95th	0.5	No	Container	Bottled	Yes	Stove	Concrete	Yes	Yes	Yes
19	1/05/16	39	Vaginal	F	15th	1	No	Cistern	Bottled	Yes	Stove	Concrete	Yes	Yes	No

a M, male; F, female.

A total of 97 stool samples were processed to search for DNA and RNA viruses by random, next-generation sequencing using the NextSeq500 Illumina platform. We obtained an average of 21,723,459 sequence reads per sample; after quality control and preprocessing, an average of 4,053,076 sequence reads per sample remained for subsequent analysis (Table S1 in the supplemental material). Within the complex virome detected, one striking observation was the common presence of caliciviruses even in the samples collected very early in the life of the infants. Norovirus was the most abundant species among the detected fecal eukaryotic viruses, representing about 32% of the total sequence reads, while sapovirus sequences represented 3.8% of the reads. In this work, we describe our findings related to the prevalence and genetic diversity of both norovirus and sapovirus strains in the population studied. A subanalysis of the viral diversity of 3 children has been published in Taboada et al. (12), and a description of the complete set of fecal viruses found in this work will be published elsewhere.

### Caliciviruses are frequently found in the gastrointestinal tract of infants throughout their first year of life.

**FIG 2:**
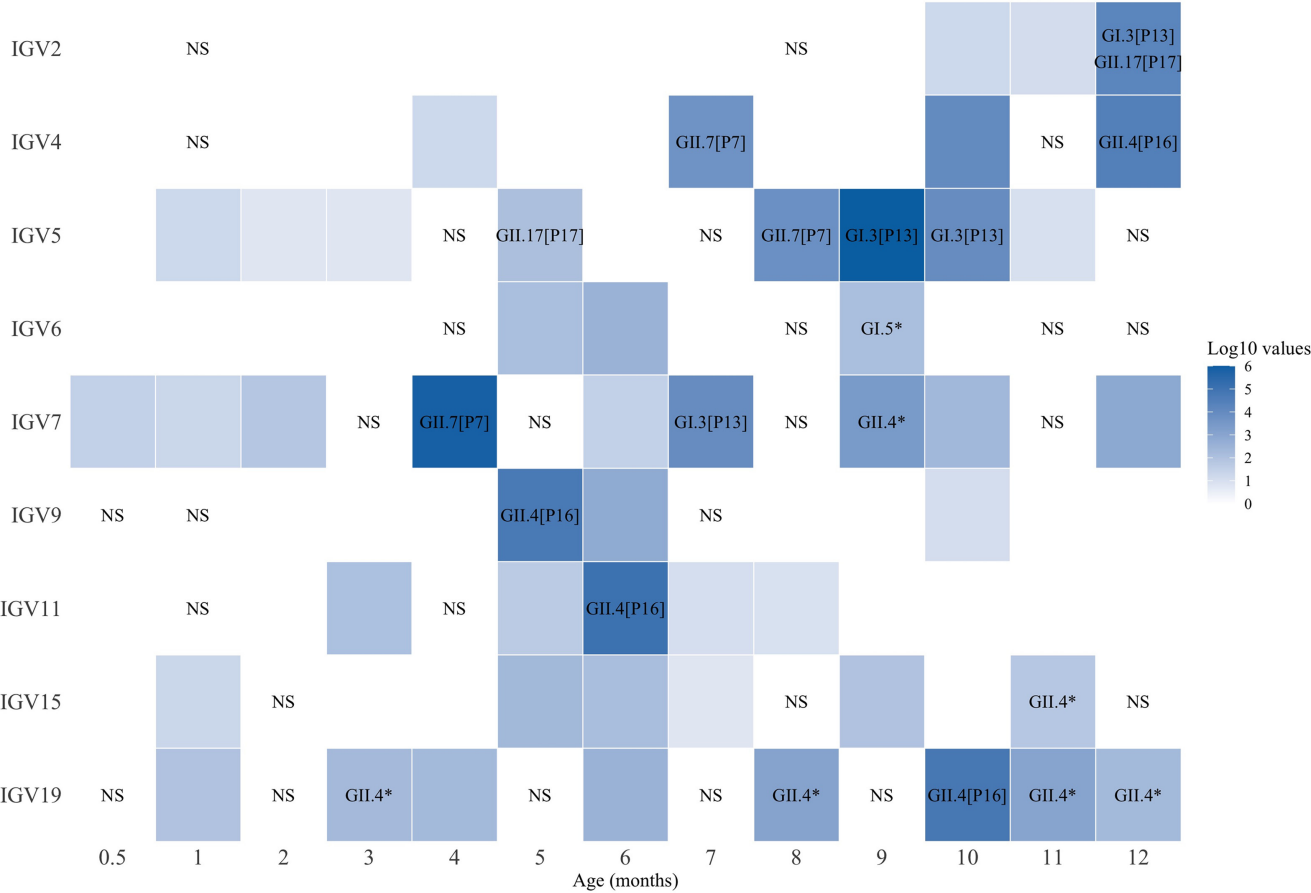
Norovirus-positive stool samples. The abundance of sequence reads was normalized to 5 million to eliminate differences in sequencing depth. Also, to simplify the figure, we added up the abundance of reads when they were present in samples collected within the same month. Values are presented as log_10_ of the normalized read abundance. Blank squares represent norovirus-negative samples; NS, no sample taken. An asterisk (*) indicates where dual genotyping could not be determined.

**FIG 3:**
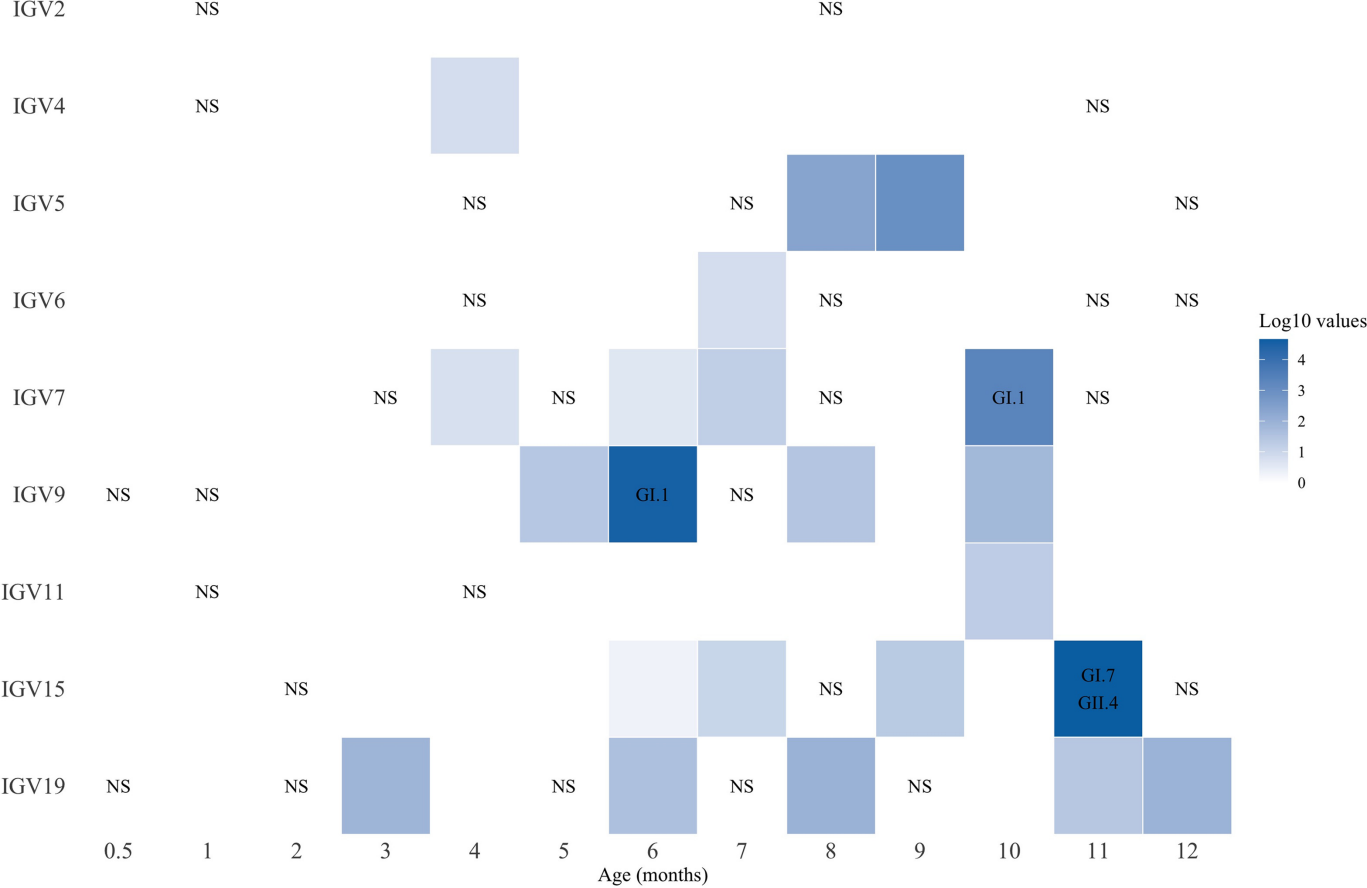
Sapovirus-positive stool samples. Sequence read abundance was normalized to 5 million reads to eliminate differences in sequencing depth. Also, to simplify the figure, we added up the abundance of reads when they were present in samples collected within the same month. Values are presented as log_10_ of the normalized read abundance. Blank squares indicate sapovirus-negative samples; NS, no sample taken.

Calicivirus sequences were detected in 58 of the 97 (60%) gastrointestinal samples analyzed, and in three children (infants 5, 7, and 19), more than 80% of their fecal samples collected across the year had reads assigned to these viruses. Of interest, caliciviruses were detected in one infant (infant 7) just 2 weeks after birth and in three other children (infants 5, 15, and 19) by 1 month of age (Fig. 2). All children were breastfed, and in all cases, calicivirus sequence reads were detected after the ingestion of liquids (e.g., water or tea) or food different from breastmilk, with the exception of infants 5 and 11 for which caliciviruses were detected when they were exclusively on a breastmilk diet. Of note, the number of sequence reads specific for caliciviruses was rather low during the first 3 months of life, making it difficult to establish if these cases represent a possible infection or passive ingestion of the virus or viral genetic material. The abundance of calicivirus sequence reads increased after the fourth month of life (Wilcoxon signed rank, *P* = 0.016) and up to 12 months of age, suggesting an augmented level of interaction of infants with potential fomites and in general with the environment, although the increment could also be related with the waning of maternally acquired immunity. At the species level, noroviruses were present in 54 (56%) of the samples (Fig. 2), while sapoviruses were found in 24 of samples (25%) (Fig. 3). The samples in which at least 20% of coverage of the complete genome was obtained were arbitrarily considered potential infections; thus, 28 (29%) and 5 (5%) samples for norovirus and sapovirus, respectively, would correspond to virus infections (Tables S2 and S3). Using this criterion, all children would have had at least one and up to six possible norovirus infections and one or two possible sapovirus infections. It is noteworthy that despite the frequent detection of caliciviruses, none of the infants showed gastrointestinal symptoms during the study; this was true even in those cases where two different norovirus genotypes were identified in the same sample or potential coinfections with norovirus and sapovirus were found (Table 2).

**TABLE 2:** Dual genotyping of consensus sequences

Genus^*a*^	Child	Age (mo)	Mo of sampling	Yr of sampling	Genotype	B region^*c*^	C region	Reference sequence length	No. of N	Base pairs in consensus
										
NoV	IGV2	12	March	2016	GI.3[P13]	93%	96%	7,556	135	7,421
		13	April	2016	GII.17[P17]	99%	99%	7,470	86	7,384
	IGV4	7	November	2015	GII.7[P7]	97%	97%	7,374	0	7,374
		11.5	March	2016	GII.4[P16]	98%	99%	7,500	1	7,499
	IGV5	4.5	September	2015	GII.17[P17]	-	71%	7,559	1,638	5,921
		7.5	December	2015	GII.7[P7]	97%	97%	7,374	0	7,374
		9	February	2016	GI.3[P13]	93%	96%	7,691	0	7,691
		10	March	2016	GI.3[P13]	93%	96%	7,572	119	7,453
	IGV6	8.5	March	2016	GI.5^*b*^	-	85%	7,701	6,368	1,333
	IGV7	3.5	November	2015	GII.7[P7]	97%	98%	7,374	0	7,374
		7	March	2016	GI.3[P13]	93%	96%	7,691	0	7,691
		8.5	April	2016	GII.4^*b*^	-	94%	7,501	188	7,313
	IGV9	4.5	April	2016	GII.4[P16]	98%	99%	7,456	45	7,411
	IGV11	5.5	April	2016	GII.4[P16]	98%	99%	7,467	34	7,433
	IGV15	11	November	2017	GII.4^*b*^	-	70%	7,501	3,608	3,893
	IGV19	3	August	2016	GII.4^*b*^	-	74%	7,502	3,420	4,082
		8	January	2017	GII.4^*b*^	-	70%	7,502	2,497	5,005
		10	February	2017	GII.4[P16]	98%	99%	7,502	23	7,479
		10.5	March	2017	GII.4^*b*^	-	86%	7,502	1,109	6,393
		11.5	April	2017	GII.4^*b*^	-	81%	7,502	3,543	3,959
										
SaV	IGV7	9.5	May	2016	GI.1	-	99%	7,375	13	7,362
	IGV9	5.5	May	2016	GI.1	-	99%	7,386	2	7,384
	IGV15	10.5	November	2016	GI.7	-	96%	7,131	321	6,810
		11	December	2017	GII.4	-	97%	7,446	14	7,432

a NoV, norovirus; SaV, sapovirus.

b Dual genotyping could not be determined due to lack of coverage of B region.

c -, no data.

### Calicivirus diversity in the study population.

The use of consensus sequence genomes and genotyping with the human calicivirus genotyping tool (14) allowed us to establish genogroups and genotypes. In norovirus, genogroup GII was the most frequently found, being identified in 15 samples, followed by GI in 6 samples. We observed the presence of at least 5 different norovirus genotypes circulating in the community, corresponding to GI.3[P13] (4 samples), GI.5 (1 sample), GII.4[P16] (10 samples), GII.7[P7] (3 samples), and GII.17[P17] (2 samples) (Table 2), with GII.4[P16] being the most prevalent. Two of the infants (2 and 4) were infected with two different norovirus genotypes during the year of study, and two infants (5 and 7) were infected with three genotypes (Table 2). This shows that multiple infections with unrelated genotypes are common. Interestingly, in child 2, two different norovirus genotypes (GI.3[P13] and GII.17[P17]) were detected in the same sample, suggesting a coinfection. However, in child 19, a GII.4 virus was detected in three consecutive samples (Fig. 2), suggesting a chronic infection with prolonged shedding of the virus, as has been reported previously (15–17). Likewise, child 5 had two consecutive samples genotyped as GI.3[P13], which could also reflect a chronic infection. Despite the low number of viruses for which the genotype could be determined, it appears that some predominance in the circulating genotype existed with time. For example, all samples of genotype GII.7[P7] were detected between November and December 2015, while all samples of genotype GI.3[P13] were found between February and March 2016 (Table 2). Sapoviruses were identified as belonging to genotypes GI.1 (2 samples), GI.7 (1 sample), and GII.4 (1 sample) (Table 2; Fig. 3). GI.1 was present in two different children (7 and 9) during the same month (May 2016), while child 15 had two different genotypes (GI.7 and GII.4) detected in the same sample.

### Calicivirus infections seem to be more frequent in children with lower-than-average weight.

Four of the studied children (infants 4, 5, 7, and 19) had an average weight corresponding to, or below, the 25th percentile (Table 1). The frequency of positive norovirus samples was significantly higher in these children than in infants in the 50th or higher weight percentile (71% versus 44% positive rates, respectively, *χ*^2^, *P* = 0.007). Likewise, the abundance of sequence reads was also significantly different (mean rank 59 versus 39, Mann-Whitney U test, *P* = 0.001) when these two groups were compared. When potential active infections, according to our definition, were considered, the same tendency was observed (mean rank 55 versus 43, Mann-Whitney U test, *P* = 0.017). Moreover, genotyping of the infecting virus could be determined in two and up to five different samples in each low-weight child (Fig. 2; Table 2). Of interest, children 5 and 19 who had a possible chronic infection, as described in the previous section, are below the 15th weight percentile. Although this low weight cannot be considered a pathological state, these observations are similar to those reported in malnourished children (18). No other significant differences were found, neither for norovirus nor sapovirus, when other factors (Table 1) were evaluated.

## DISCUSSION

In this study, we describe the prevalence and diversity of both noroviruses and sapoviruses detected by random next-generation sequencing as part of a metagenomic study designed to determine the fecal virome of apparently healthy children, with monthly sampling during their first year of life.

Many studies have characterized the presence of caliciviruses in children hospitalized with acute gastroenteritis, but few have been carried out in community settings, and even fewer have been designed as longitudinal studies. In a large follow-up study of 1,457 children performed in 8 low- and middle-income countries, stool samples were routinely collected monthly from age 1 to 12 months and at 15, 18, 21, and 24 months of age (19); a prevalence of 19% norovirus asymptomatic carriage was observed in a subset of 199 children (18). Similarly, a meta-analysis of Latin American studies (2) and a study conducted in Mexico (10) described prevalences of norovirus asymptomatic cases in community settings of 8% and 7%, respectively. Also, a meta-analysis that included 81 countries described a global prevalence of 7% in community settings (20). In this study, we found sequence reads related to norovirus in at least 2 and in up to 10 stool samples collected across the year for each child, representing a total of 54 (56%) positive samples.

As for sapoviruses, a longitudinal study in Perú (21) performed in 100 children followed for up to 2 years of age showed a reverse transcription-quantitative PCR (RT-qPCR) prevalence of 9.7% in nondiarrheal samples. As in other studies (22), the incidence of sapovirus was higher during the second year of life (1.33 cases per child/year) than in the first year (0.51 cases per child/year), and 82% of the children had at least one sapovirus infection by the second year, 64% of which were associated with diarrhea. In our study, 24 (25%) positive samples were associated with sapovirus sequences, a lower proportion than that observed for noroviruses, possibly reflecting their higher frequency of infection after the first year of life, as described in reference 22, and not necessarily a bias of sequencing depth.

The percentage of samples positive for calicivirus sequences in the present study, as detected by next-generation sequencing, was considerably higher (norovirus, 56%; sapovirus, 25%) than that reported in studies that have used RT-qPCR for virus detection in community settings in both Mexico and globally (2, 10, 18–23). If we consider the more stringent criterion of at least 20% genome coverage to represent active infections, our results (norovirus, 29%; sapovirus, 5%), although higher, match more closely with what has been reported in other studies. With this criterion, all children would have had at least one asymptomatic norovirus infection (3 cases per child/year), and 3 children would have had one asymptomatic sapovirus infection (0.33 cases per child/year). We believe that this rate of detection is related to the higher sensitivity of the next-generation sequencing method used that could detect virus that is passing through but does not result in infection. However, although RT-qPCR is considered the “gold standard” for norovirus detection, difficulties in interpretation of positive results in asymptomatic individuals have been raised (24, 25). In contrast, next-generation sequencing methods are known as state of the art in the detection of viruses, with the cost of this technology being the main disadvantage. Furthermore, because RT-qPCR only targets open reading frame 1 (ORF1) and ORF2 (25), this could compromise the description of uncommon genogroups.

In this study, a large diversity of norovirus strains was identified. In agreement with previous studies (17, 18, 26), genotype GII.4 was the most frequently found, being present in 66% of the children. GI.3 and GII.7 genotypes were also identified in 33% of the children, including two children (5 and 7) who had both genotypes at different time points. Genotype GI.3 has been associated with waterborne outbreaks (27, 28), suggesting that contaminated water could have been an important source of caliciviruses during this study, especially since the water in the community is usually obtained from a water well. However, sustained breastfeeding beyond 6 months of age could have helped for the low frequency of digestive and respiratory symptoms in these children, something we have found in previous studies in this community (29). In addition, our findings agree with previous observations that indicate that norovirus reinfections are common with not closely related genotypes (17, 18, 21, 22, 26), in accordance with a proposed model that clusters genotypes into immunotypes and suggests that protection between close genotypes might exist (30).

In a Peruvian birth cohort study, it was reported that multiple norovirus infections can be observed, with asymptomatic infections being common (46% of cases) (17). These infections were significantly higher in infants younger than 6 months of age than in those aged 6 to 11 months; exclusive breastfeeding during age 3 to 5 months was found to be protective (17). In our study, we found a higher frequency of positive samples and a higher abundance of sequence reads after the fourth month of life. The low calicivirus frequency and amount of reads in samples taken before this time may represent infections “aborted” or restricted by calicivirus antibodies present in the mothers’ milk, as previously suggested (17), or may be the consequence of a transient infection that was unable to colonize a still immature intestinal epithelium.

The limitations of our study include the reduced number of children included, which is not representative of the population, making it difficult to draw firm conclusions beyond those presented in this report; the withdrawal of 11 of the 20 infants initially enrolled particularly affected this aspect of the study. However, it is important to point out that the monthly sampling produced a total of 97 fecal samples, which, although still limited in number, allowed us to describe a year of calicivirus infection in asymptomatic children. Also, and paradoxically, the high sensitivity of our metagenomic analysis complicates the clinical interpretation (infection versus carriage) of the presence of sequence reads in the stool samples, especially when they are in low numbers.

This study shows the value of metagenomics to explore in more detail the prevalence and dynamics of viral gastrointestinal infections. It reveals more closely the initial interaction of viruses with the infant gut, whether they represent real infections or asymptomatic carriage. We are interested in determining the source of viruses that can be detected in the early months of life, which may hint at how the gut eukaryotic virome of infants is acquired.

## MATERIALS AND METHODS

### Sampling.

This study was conducted in Xoxocotla, Morelos, a semirural community located 40 km south of the city of Cuernavaca; thus, even though it is a semirural town, it maintains a connection with the capital city of the State of Morelos. The work was performed in collaboration with the Ministry of Health of the State of Morelos. Healthy women who were in the last trimester of pregnancy and who had attended maternity health services at the community Health Center were invited to participate. Stool samples from newborns were collected starting 15 days after delivery and monthly thereafter until the infants were 12 months of age. During this time, height, weight, food intake, and daily symptoms were registered. Inclusion criteria for children included singleton delivery, pregnancy at term, clinically healthy, and no congenital diseases. To collect fecal samples from diapers, sterile plastic containers were used; the diapers were used inverted to avoid absorption of the samples. The samples were maintained at 4°C on the day of collection, kept at −20°C for 1 week at the village laboratory, and finally transported to the Institute of Biotechnology-National University of Mexico (UNAM) where they were stored at −70°C until use. None of the mothers reported illness symptoms in their children during the study period. It is important to mention that the analysis of caliciviruses presented in this study is a subanalysis of a larger metagenomic project that will be published elsewhere; 20 participants were originally enrolled, but due to incomplete sample collection, 11 were eliminated from the analysis. Only children with at least 80% of the planned samples collected were included.

### Nucleic acid isolation and sequencing.

Nucleic acids were extracted essentially as described previously (31, 32), and the sequencing library was prepared by a random approach (33, 34). Briefly, RNA was reverse transcribed using SuperScript III reverse transcriptase (Invitrogen, USA) using primer A 5′-GTTTCCCAGTAGGTCTCN9-3′. This primer contains nine random nucleotides at the 3′-end to allow random priming of cDNA synthesis. The second cDNA strand was generated with the same primer A using Sequenase 2.0 (USB, USA) for two rounds of synthesis. Two rounds of synthesis with Sequenase 2.0 allows for the generation of DNA fragments having a terminal primer A sequence, also from DNA purified templates, which are not transcribed by reverse transcriptase. At the last step, the cDNA was amplified with Phusion high-fidelity polymerase (Finnzymes) using primer B 5′-GTTTCCCAGTAGGTCTC-3′, homologous to a specific portion of primer A, for 10 additional cycles. This step allowed amplification of only DNA fragments previously generated by primer A. The amplified DNA was cleaned with a ZYMO DNA clean and concentration 5 kit. Sequencing libraries were prepared using a Nextera XT DNA library preparation kit (Illumina). Samples were tagged, pooled, and deep sequenced on a NextSeq500 Illumina platform, delivering paired-end reads of 75 bp. Base calling was performed using Illumina Real Time Analysis v1.18.54, and demultiplexing was performed using bcl2fastq v2.15.0.4.

### Sequence analysis of viral genomes.

Every sample was preprocessed, including quality control (35), removal of duplicates (36), and human and ribosomal filtering, as previously described (33). The valid reads were then mapped using BBMap v38.26 (37) against a database of all complete genomes of viruses in the *Calicivirus* family reported in GenBank by May 2020 (38) and reduced at 88% identity with Cd-Hit v4.8.1 (36). Mapped reads at species level were used to perform *de novo* assembly using IDBA v1.1.3 (39) or SPADES v3.13.0 (40). Consensus sequences were generated in those cases in which complete genomes were not obtained by mapping the contig to a reference using Bowtie2 v2.3.4.3 (41). Next, Samtools v1.9 (42) was used to obtain a first consensus sequence. Consensus calling was performed with iVar v1.3.1 (43), using a base score of a Q value of >20 and a minimum read coverage depth of 2× to call a base and “N” for lower values. For single nucleotide polymorphisms (SNP) calling, a threshold of majority base rule was used with a minimum of 10× coverage. Each consensus or complete genome was verified using BLAST v2.7.1 (44). To detect the polymerase in each consensus sequence, we used the web-based Human Calicivirus Typing tool (14). The remaining reads were taxonomically assigned by blastn and then analyzed using MEGAN v6.19.9 (45). All samples were normalized for statistical analysis and figures to adjust for differences in sequencing depth. For this, we divided the number of viral reads by the valid reads of each sample and normalized the values to a depth of 5 million reads per sample.

### Statistical analysis.

Variables that compared different groups of children were analyzed with a Mann-Whitney U test (two tailed). Variables that compared time points (before and after) were analyzed with a Wilcoxon signed rank test. Categorical variables were compared using a *χ*^2^ test in R v3.6.3 (46). Statistical analysis was carried only in samples taken during the first year of life.

### Ethical considerations.

The protocol used in this study was conducted under the ethical principles and approval of both the Mexican Ethics and Research Commission of the Health Ministry of the State of Morelos and the Ethics in Research Commission of the Faculty of Medicine (project number 088/2014) of the National University of Mexico (UNAM). It was also approved by the Bioethics Committee of the Institute of Biotechnology (project number 261) of UNAM. The guidelines of the Committees are based on the Mexican Official Norm (Norma Oficial Mexicana NOM-012-SSA3-2007) that regulates the ethical principles of research on humans as well as on the Declaration of Helsinki regarding research studies involving humans, approved by the World Health Organization. Written informed consent was obtained from each parent or guardian prior to enrollment.

### Data availability.

Sample raw sequences are available at BioProject under accession number PRJNA592261 at NCBI. Complete genomes for norovirus were submitted (SUB9919751) with the following accession numbers G2_12_MAR16 (MZ462929), G5_9_FEB16 (MZ462930), G5_10_MAR16 (MZ462931), G7_7_MAR16 (MZ462932), and G15_13.5_FEB17 (MZ462933) and at SUB9919784 with the following accession numbers G2_12_MAR16 (MZ478134), G4_7_NOV15 (MZ478135), G4_11_MAR16 (MZ478136), G5_7.5_DIC15 (MZ478137), G7_3_NOV15 (MZ478138), G9_4.5_ABR16 (MZ478139), G11_6_ABR16 (MZ478140), and G19_10_FEB17 (MZ478141). For the sapovirus genome, the accession number is G15_10.5_NOV16 (MZ488271).
